# Baseline ^18^F-FDG PET/CT habitat radiomics versus dual-channel deep learning for predicting interim PET early metabolic response in diffuse large B-cell lymphoma: a comparative study

**DOI:** 10.3389/fonc.2026.1801204

**Published:** 2026-06-04

**Authors:** Yu He, Shun Wang, Yingchun Li, Xinyang Li, Jingkai Yi, Dan Wang, Kailin Qi, Yongjiang Li, Xiao Jiang, Yutang Yao, Ping Wu, Meng Zhao, Hao Lu, Taipeng Shen, Zhuzhong Cheng, Ying Kou

**Affiliations:** 1 Medical Imaging Department, North Sichuan Medical College, Nanchong, China; 2 Department of Nuclear Medicine, Sichuan Clinical Research Center for Cancer, Sichuan Cancer Hospital & Institute, Sichuan Cancer Center, University of Electronic Science and Technology of China, Chengdu, China; 3 Department of Nuclear Medicine and Radiotherapy, Chinese People’s Liberation Army Western Theater Command Air Force Hospital, Chengdu, Sichuan, China; 4 Department of Medical Oncology, Sichuan Clinical Research Center for Cancer, Sichuan Cancer Hospital & Institute, Sichuan Cancer Center, University of Electronic Science and Technology of China, Chengdu, China

**Keywords:** deep learning, diffuse large B-cell lymphoma, habitat radiomics, interim PET, PET/CT

## Abstract

**Purpose:**

To develop baseline ¹^8^F-FDG PET/CT habitat radiomics and dual-channel deep learning models for the prediction of early metabolic response (EMR) on interim PET (iPET) in patients with DLBCL and compare their performance.

**Methods:**

Patients with DLBCL who underwent baseline ^18^F-FDG PET/CT between December 2018 and August 2024 and received 2–4 cycles of R-CHOP or R-CHOP-like chemotherapy were retrospectively enrolled. Based on iPET Deauville scores, patients with scores of 1–3 were classified as EMR, whereas those with scores of 4–5 were classified as non-EMR. Lesions were semi-automatically segmented to generate volumes of interest (VOIs). Voxel-wise habitat subregions were delineated using K-means clustering to characterize intratumoral heterogeneity. Radiomics features were extracted from whole-tumor and habitat subregions on PET and CT images. For deep learning, the largest axial tumor slice from PET and CT was concatenated along the channel dimension as dual-channel input. Model performance was evaluated using the area under the receiver operating characteristic curve (AUC), accuracy, balanced accuracy, sensitivity, specificity, and F1 score. Calibration curves, decision curve analysis, net reclassification improvement, and integrated discrimination improvement were additionally used for model comparison.

**Results:**

A total of 148 patients (101 EMR, 47 non-EMR) were randomly split into training (n = 103) and test (n = 45) sets. This retrospective single-center study used the training set for feature selection and model development, and the test set exclusively for final evaluation. We developed habitat radiomics and dual-channel deep learning models; multilayer perceptron and DenseNet-161 were selected as the optimal architectures, respectively. In the test set, the habitat radiomics model (Habitat_MLP) achieved an AUC of 0.871 (95% CI: 0.7563–0.9857), with a specificity of 0.903, and accuracy of 0.822, showing higher overall performance than the dual-channel deep learning model (DL_DenseNet161), which achieved an AUC of 0.793 (95% CI: 0.6409–0.9444), specificity of 0.677, and accuracy of 0.711. Furthermore, the Habitat_MLP model showed more favorable calibration, higher net benefit on DCA, and improved risk reclassification in this dataset.

**Conclusion:**

The habitat radiomics model derived from baseline ^18^F-FDG PET/CT (Habitat_MLP) demonstrated superior overall performance and robustness for predicting EMR on iPET in patients with DLBCL, suggesting its potential value as a decision-support tool for pretreatment risk stratification.

## Introduction

Diffuse large B-cell lymphoma (DLBCL) is an aggressive B-cell–derived non-Hodgkin lymphoma (NHL) and accounts for approximately 25%–30% of all NHL cases, making it the most common histologic subtype (1, 2). Rituximab-based chemoimmunotherapy, most commonly R-CHOP or R-CHOP-like regimens, remains the standard first-line treatment for DLBCL and has substantially improved outcomes. Nevertheless, approximately 30%–40% of patients still experience inadequate response or early disease progression after initial therapy (3, 4). Therefore, early identification of patients at high risk of poor treatment response, enabling effective risk stratification and risk-adapted management, remains an important clinical need in the management of DLBCL.

^18^F-FDG PET/CT plays a central role in disease staging, response assessment, and prognostic evaluation in DLBCL (5). Interim PET (iPET) is recommended by the Lugano classification and the Deauville 5-point scale is widely used for early response evaluation. Deauville scores of 1–3 are commonly considered iPET-negative and indicative of early metabolic response (EMR), whereas scores of 4–5 are regarded as iPET-positive (non-EMR) and are associated with progression-free and overall survival (6–8). However, iPET is performed during therapy and therefore provides limited prospective guidance before treatment initiation. Accordingly, noninvasive approaches based on baseline imaging that can prospectively identify patients who are likely or unlikely to achieve EMR are clinically valuable.

Radiomics has emerged as a powerful approach for characterizing tumor heterogeneity and predicting outcomes through high-throughput extraction of quantitative imaging features (9). Multiple studies have demonstrated that radiomics features derived from ^18^F-FDG PET/CT can be used to predict treatment response and prognosis in DLBCL (10, 11). However, conventional radiomics often treats the tumor as a single homogeneous region, potentially overlooking intratumoral spatial heterogeneity in metabolism, structure, and microenvironmental states. Such whole-tumor averaging may obscure localized information relevant to treatment response (12). Habitat radiomics addresses this limitation by partitioning tumors into subregions with similar imaging characteristics using unsupervised voxel-wise clustering and extracting region-specific features to better quantify spatial heterogeneity (13, 14). In addition, deep learning, an important development direction in medical image analysis, has achieved encouraging progress in a variety of tumor imaging prediction tasks due to its end-to-end feature learning capability (15, 16). For multimodal PET/CT, multi-channel inputs can fuse representative PET and CT slices at the channel level, enabling early integration of metabolic and anatomical information and potentially improving discrimination (17, 18).

Against this background, we developed a habitat radiomics model and a dual-channel deep learning model based on baseline ¹^8^F-FDG PET/CT to predict EMR (vs. non-EMR) on iPET in patients with DLBCL. We then systematically compared their predictive performance and potential clinical utility using the same prediction task and dataset.

We present this article in accordance with the TRIPOD reporting checklist.

## Materials and methods

### Study design

**Figure 1:**
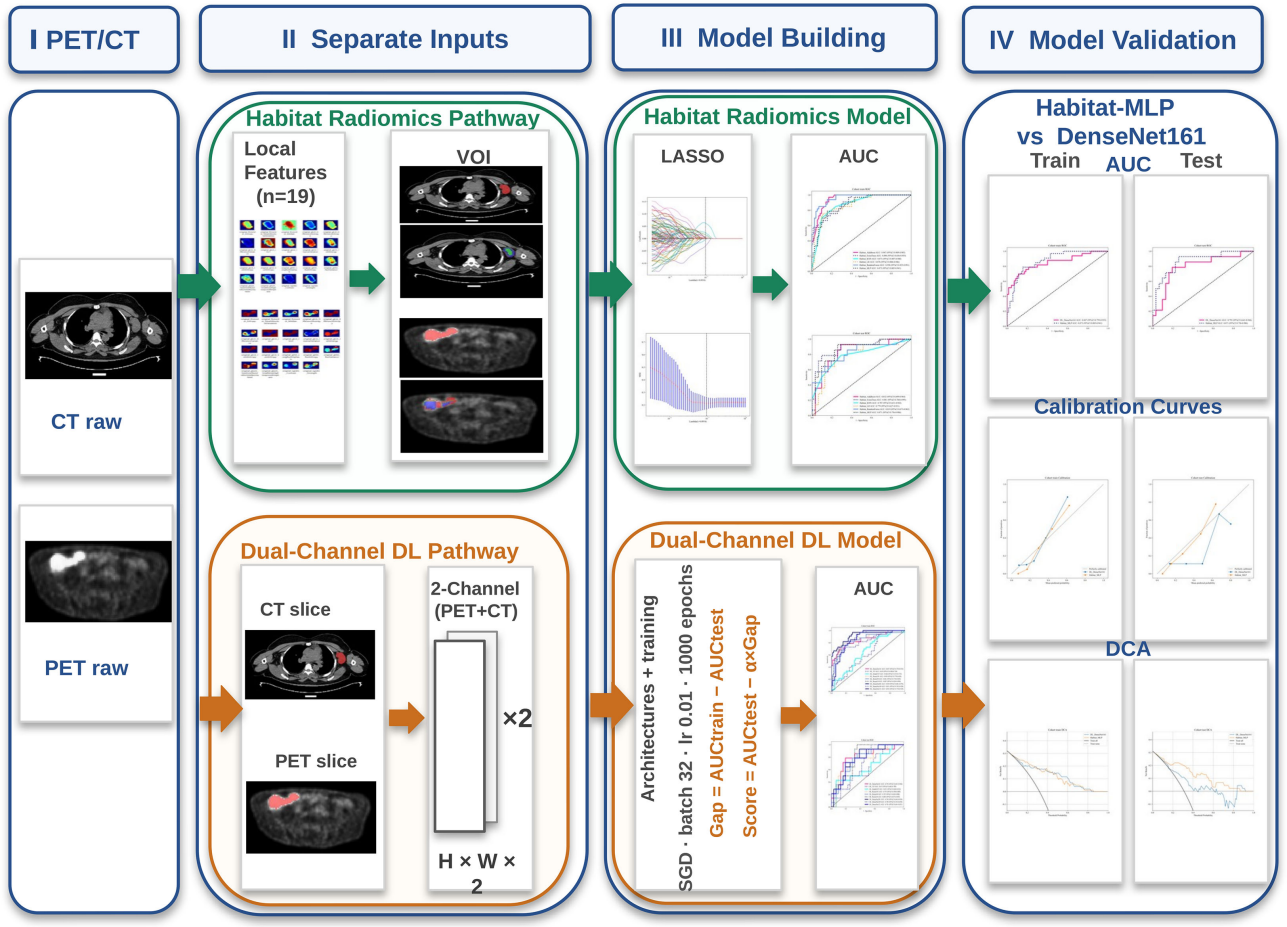
The workflow of this study. CT, computed tomography, PET, positron emission tomography, VOI, volume of interest, LASSO, least absolute shrinkage and selection, AUC, area under the receiver operating characteristic curve, DCA, decision curve analysis.

This study developed a habitat radiomics model and a dual-channel deep learning model based on PET/CT inputs to predict EMR status (EMR vs non-EMR) on iPET in patients with DLBCL. The study workflow is shown in **Figure 1**.

### Patient data

**Figure 2:**
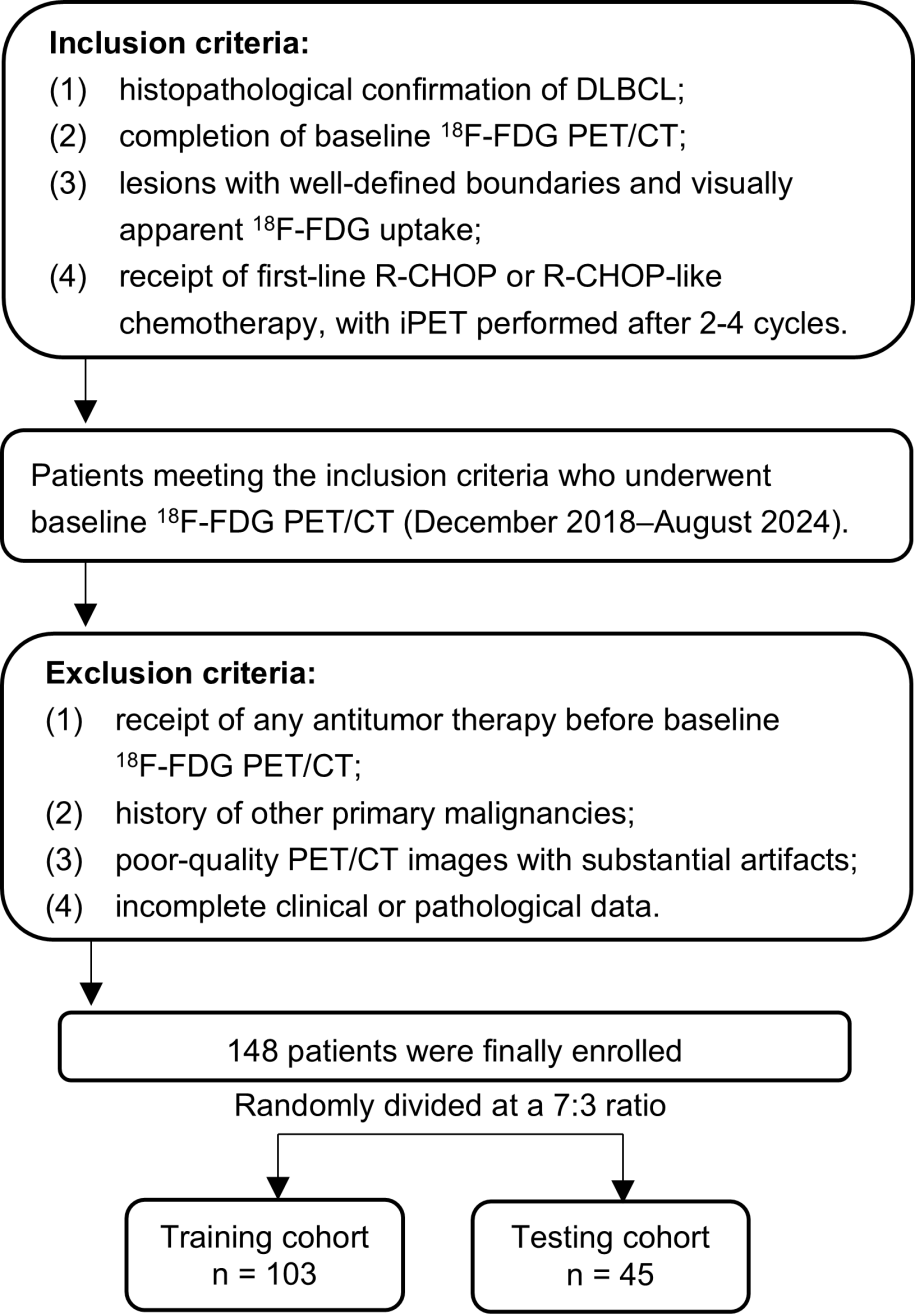
Flowchart depicting the enrolled patients in the study.

This retrospective study included patients with pathologically confirmed DLBCL who underwent baseline ¹^8^F-FDG PET/CT at our center between December 2018 and August 2024. The inclusion and exclusion criteria are summarized in **Figure 2**. Clinical variables were recorded, including age, sex, cell-of-origin (COO) subtype, Ann Arbor stage, Ki-67 index, and MYC/BCL-2 dual-expression status.

This study was a retrospective analysis. Ethical approval was obtained from the institutional ethics committee (Approval No. KY-2025-373-01). Due to the retrospective nature of the study, the requirement for written informed consent was waived.

### PET/CT imaging protocol

All patients underwent PET/CT on a Siemens Biograph mCT-64 scanner (Siemens Healthineers, Germany). ¹^8^F-FDG was produced using a Sumitomo HM-10HC medical cyclotron and synthesis module (Sumitomo Heavy Industries, Japan), with radiochemical purity >99%. Patients fasted for at least 6 hours, and fasting blood glucose was confirmed to be <11.1 mmol/L before tracer injection. ¹^8^F-FDG was administered intravenously at 3.7–5.5 MBq/kg, followed by an uptake period of approximately 60 minutes at rest. A low-dose CT scan for attenuation correction and anatomical localization was acquired first (120 kV, 80 mAs). PET emission data were then acquired for 1.5 minutes per bed position for the torso (typically 6–7 beds) and 2.0 minutes per bed position for the head. Images were reconstructed using the TrueX + time-of-flight (TOF) ultraHD-PET algorithm (torso: 3 iterations/21 subsets; head: 4 iterations/25 subsets). PET/CT fusion and interpretation were performed immediately after acquisition. The specific models and manufacturers of the PET/CT scanner and cyclotron are provided in **Supplementary Material 1**.

### Image evaluation

Interim PET (iPET) response was evaluated according to the Deauville 5-point scale. Patients with Deauville scores of 1–3 were classified as having an early metabolic response (EMR), whereas those with Deauville scores of 4–5 were classified as non-EMR. The dichotomized EMR/non-EMR classification was used as the clinical reference standard for model development and performance evaluation in this study.

All iPET Deauville scores were assigned following the routine imaging interpretation and reporting workflow at our institution. Each iPET scan was first visually assessed by a physician with extensive experience in PET/CT interpretation and was subsequently reviewed by a senior imaging physician as part of the standard clinical reporting process. This double-reading workflow was used to improve the consistency and reliability of Deauville-based response assessment.

In addition to the routine clinical review process, an independent blinded assessment was performed during research data preparation. Specifically, two nuclear medicine physicians, each with more than 10 years of experience in clinical PET/CT interpretation, independently reviewed all iPET images and assigned Deauville scores. During image evaluation, both readers were blinded to patients’ clinical outcomes and other research-related information. Any discrepancies in Deauville scoring were resolved through consensus discussion, and the final consensus results were used to define EMR status.

### Volume of interest delineation and image preprocessing

PET images were imported into LIFEx (version 7.6.0; https://lifexsoft.org) in DICOM format. All identifiable hypermetabolic nodal and extranodal lesions in each patient were semi-automatically delineated using a 40% SUVmax threshold. The lesion masks were then combined to form a single composite tumor VOI per patient for subsequent feature extraction, followed by manual exclusion of regions with physiological uptake and adjacent non-tumor tissues. The initial VOI delineation and preliminary editing were performed by an intermediate nuclear medicine physician with 8 years of experience. All VOIs were subsequently reviewed and refined by a senior nuclear medicine expert (chief physician) with >15 years of experience, with special attention to physiological ¹^8^F-FDG activity in the urinary system (e.g., kidneys/collecting system and urinary bladder). Both readers were blinded to EMR status during VOI delineation and verification. Corresponding CT images acquired on the same scanner were imported in DICOM format. To minimize potential PET/CT misregistration caused by respiratory motion or patient movement, CT images were rigidly registered to the corresponding baseline PET images using ITK-SNAP before VOI mapping and feature extraction. After registration, PET/CT alignment quality was visually inspected on fused images in axial, coronal, and sagittal planes, with particular attention to the spatial correspondence between FDG-avid lesions and corresponding CT anatomical structures. For cases with minor residual mismatch, lesion boundaries and VOI placement were carefully reviewed and refined with reference to both PET metabolic uptake and CT anatomical information. Regions with physiological uptake or adjacent non-tumor tissues were manually excluded when necessary. If severe misregistration precluded reliable lesion localization or VOI mapping, the case was excluded. The PET-derived VOIs were then mapped onto the registered CT images in 3D Slicer (version 5.6.1; https://www.slicer.org) to obtain the CT VOI regions. In addition, PET semi-quantitative parameters were calculated and collected using LIFEx (version 7.6.0), including maximum standardized uptake value (SUVmax), metabolic tumor volume (MTV), standardized metabolic tumor volume (sMTV), total lesion glycolysis (TLG), standardized total lesion glycolysis (sTLG), and the maximum distance between lesions (Dmax). To standardize voxel spacing, both PET and CT images were resampled to a fixed spatial resolution of 1 × 1 × 1 mm using nearest-neighbor interpolation.

### Habitat clustering and habitat radiomics feature extraction

Subregional habitats refer to imaging-defined intratumoral subregions generated by unsupervised voxel-wise clustering of standardized multi-parametric local feature vectors. Voxels assigned to the same cluster share similar local metabolic and textural patterns and are mapped back to the tumor VOI to form a spatially coherent habitat subregion. To construct these feature vectors, a moving window technique was first applied to compute the local entropy of PET and CT images within a 5 × 5 × 5 voxel neighborhood. Subsequently, to ensure complete feature extraction at the boundaries, each VOI was expanded outward by two voxels. Finally, a 19-dimensional local feature vector was constructed for each voxel within the VOI by integrating multiple quantitative parameters. A detailed summary of these 19 features is provided in **Supplementary Material 2**.

After feature standardization, unsupervised clustering was performed using the K-means algorithm with Euclidean distance as the similarity metric. In this study, K-means clustering was performed using a global cohort-level clustering strategy rather than independently within each individual patient VOI. Specifically, standardized voxel-wise 19-dimensional local feature vectors derived from tumor VOIs were pooled into a common feature space, and K-means clustering was applied to identify shared imaging-defined habitat patterns across the cohort. The resulting cluster centroids served as a unified definition of habitat categories, and voxels from each patient were assigned to the corresponding habitat according to their similarity to these global centroids. This strategy was used to ensure that the same habitat label represented a comparable imaging phenotype across different patients.

The number of clusters (K) was evaluated over a range from 3 to 10. The optimal clustering configuration was primarily determined using the Calinski–Harabasz index, with the Silhouette score and Davies–Bouldin index used as additional reference metrics. A higher Calinski–Harabasz index and Silhouette score, together with a lower Davies–Bouldin index, were considered to indicate better cluster compactness and separation.

Following Image Biomarker Standardisation Initiative (IBSI) guidelines, radiomics features were extracted from the whole-tumor region and habitat subregions using the PyRadiomics package (http://pyradiomics.readthedocs.io). Because of tumor heterogeneity, interpatient variability in tumor volume, and differences in internal tumor architecture, habitat subregions were absent in some cases. In addition, in a small number of patients with relatively small tumor volumes or atypical imaging patterns, some predefined habitats failed to form valid spatial clusters after K-means clustering, resulting in missing features. To retain these cases and preserve data completeness, missing values were imputed using the K-nearest neighbors (KNN) method (K = 5). KNN imputation was selected because it estimates missing values based on samples with similar multivariate radiomics profiles, which is consistent with the high-dimensional and interrelated nature of habitat radiomics features. This approach helps preserve patient-level similarity and the internal structure of the feature space. The value of K was set to 5 to balance local-neighbor information and imputation stability. To prevent information leakage, all data preprocessing and feature selection procedures were strictly confined to the training set. Specifically, the mean and standard deviation used for Z-score normalization were derived exclusively from the training set and subsequently applied to the independent test set for standardization. Subsequent feature screening—including independent-samples t-tests (P ≤ 0.05) and redundancy filtering via Pearson correlation (r > 0.9)—was performed solely using the training data. Finally, the Least Absolute Shrinkage and Selection Operator (LASSO) regression was applied to the normalized training features, with the optimal regularization parameter λ determined via 10-fold cross-validation. Following feature selection, multiple widely used machine learning algorithms, including logistic regression (LR), K-nearest neighbors (KNN), random forest (RF), extremely randomized trees (Extra Trees), multilayer perceptron (MLP), and AdaBoost, were used to construct habitat radiomics prediction models. Model selection and parameter tuning were performed within the training set, and the independent test set was used only for final performance evaluation.

### Training of the dual-channel deep learning models

For each patient, we extracted the axial slice with the largest tumor cross-section from PET and CT and stacked them along the channel dimension to form a dual-channel PET/CT input. This dual-channel configuration represents an early fusion strategy, specifically image-level/channel-level fusion, in which PET and CT information was integrated at the input stage rather than through separate modality-specific branches. Using this input configuration, multiple deep learning models based on widely used convolutional neural network (CNN) and vision transformer architectures were constructed and trained to compare the feature-learning capabilities of different network designs. The evaluated architectures included the ResNet family (ResNet18, ResNet50, and ResNet101), the DenseNet family (DenseNet121, DenseNet161, DenseNet169, and DenseNet201), and transformer-based models (Vision Transformer [ViT] and SimpleViT). All deep learning models were initialized randomly and trained from scratch without using ImageNet pre-trained weights. This strategy was adopted because the input data consisted of dual-channel PET/CT images rather than standard three-channel natural images. In particular, PET images encode tumor metabolic information, whereas CT images provide anatomical and density-related information, both of which differ substantially from the visual semantics of ImageNet natural images. Therefore, the models were trained to learn PET/CT-specific fused representations directly from the study dataset while avoiding potential domain bias introduced by natural-image pre-training. Model training was performed using the stochastic gradient descent (SGD) optimizer with a batch size of 32, an initial learning rate of 0.01, and a maximum of 1000 epochs. These hyperparameters were not optimized through exhaustive grid search, random search, or formal validation-based tuning. Instead, the same fixed training configuration was applied to all evaluated architectures to ensure a fair comparison under identical training conditions. The settings were determined based on the default configuration of the implementation framework, preliminary training stability, computational feasibility, and the limited sample size. To comprehensively evaluate both discriminative performance and generalization stability, the area under the receiver operating characteristic curve (AUC) was calculated in the training set and test set and the generalization gap was defined as Gap = AUC_train − AUC_test. A composite score was defined as Score = AUC_test − α × (AUC_train − AUC_test), where α is a penalty weight for overfitting (set to 1.0 in this study). The model with the highest composite score was selected as the optimal dual-channel deep learning architecture for subsequent comparison with the habitat radiomics model.

### Statistical analysis

Categorical variables were compared using the chi-square (χ²) test, and continuous variables were compared using independent-samples t-tests to assess baseline differences. Model predictive performance was evaluated using AUC, accuracy (ACC), balanced accuracy (BACC), sensitivity (SEN), specificity (SPE), and F1 score. Balanced accuracy was additionally used to provide a more reliable performance evaluation in the presence of class imbalance. Calibration curves were used to assess model calibration. Decision curve analysis (DCA) was performed to evaluate clinical net benefit. Net reclassification improvement (NRI) and integrated discrimination improvement (IDI) were used to compare risk stratification across models.

## Results

### Patient characteristics

This retrospective study included 148 patients with pathologically confirmed DLBCL (101 EMR [68.2%] and 47 non-EMR [31.8%]) who underwent baseline ¹^8^F-FDG PET/CT and received iPET evaluation after completing 2–4 cycles of R-CHOP or R-CHOP-like chemotherapy. Patients were randomly split with stratification by EMR status into a training set (n = 103) and a test set (n = 45) in a 7:3 ratio to maintain consistent EMR/non-EMR proportions between the two sets. Baseline characteristics, including clinical data and PET-derived metabolic parameters, are summarized in **Table 1**. To evaluate the predictive value of clinical variables and conventional PET semi-quantitative parameters for predicting EMR status on iPET, univariate logistic regression analyses were performed. Age, Ki-67 index, Ann Arbor stage, SUVmax, cell-of-origin (COO) subtype, and maximum distance between lesions (Dmax) were significantly associated with EMR status (P < 0.05). However, in stepwise multivariable logistic regression, none of these variables showed a stable independent association. Accordingly, a separate multivariate clinical prediction model was not constructed, and clinical variables were not further incorporated into the comparative analysis of imaging-based models. Detailed results of the univariable and multivariable analyses are presented in **Table 2**.

**Table 1:** Baseline characteristics of patients in cohorts.

Characteristic	Label=ALL	Label=Test	Label=Train	P-value
Age (years)	58.36 ± 13.67	57.16 ± 14.28	58.88 ± 13.43	0.493
Ki67	76.18 ± 12.29	75.67 ± 12.41	76.41 ± 12.30	0.701
TLG	2954.35 ± 3880.76	3223.55 ± 4031.62	2836.74 ± 3827.15	0.987
sTLG	52.26 ± 69.34	57.85 ± 73.90	49.81 ± 67.47	0.874
MTV	283.01 ± 349.82	333.61 ± 429.34	260.90 ± 308.59	0.822
sMTV	5.96 ± 9.71	8.69 ± 14.87	4.77 ± 4.98	0.838
Dmax	34.49 ± 27.80	36.96 ± 32.01	33.42 ± 25.84	0.96
SUVmax	24.88 ± 9.28	24.37 ± 7.36	25.10 ± 10.03	0.66
Gender				0.335
Female	75(50.68)	26(57.78)	49(47.57)	
Male	73(49.32)	19(42.22)	54(52.43)	
COO				1.0
GCB	48(32.43)	15(33.33)	33(32.04)	
Non-GCB	100(67.57)	30(66.67)	70(67.96)	
Ann_Arbor				0.996
I	11(7.43)	3(6.67)	8(7.77)	
II	56(37.84)	17(37.78)	39(37.86)	
III	29(19.59)	9(20.00)	20(19.42)	
IV	52(35.14)	16(35.56)	36(34.95)	
DEL				0.574
Negative	92(62.16)	30(66.67)	62(60.19)	
Positive	56(37.84)	15(33.33)	41(39.81)	

^†^ SUV max, maximal standardized uptake value; MTV, total metabolic tumor volume; SMTV, standardized total metabolic tumor volume; TLG, total lesion glycolysis; STLG, standardized total lesion glycolysis; D max, maximal inter-lesion distance; DEL, Double-Expressor Lymphoma.

**Table 2:** Univariate and multivariate analysis of clinical characteristics.

Feature name	OR_UNI	95% CI	P-value-UNI	OR_MULTI	95% CI	P-value-MULTI
COO	0.522	0.345-0.790	0.010	1.029	0.485-2.186	0.95
Ann_Arbor	0.828	0.738-0.928	0.007	1.2	0.766-1.878	0.503
SUVmax	0.980	0.968-0.992	0.008	1.027	0.989-1.067	0.250
Age	0.988	0.983-0.994	0.001	0.986	0.965-1.008	0.284
Dmax	0.989	0.981-0.997	0.027	1.0	0.983-1.017	0.990
Ki67	0.991	0.986-0.995	0.001	0.986	0.968-1.004	0.211
DEL	0.640	0.378-1.083	0.163			
Gender	0.687	0.436-1.084	0.176			
sMTV	0.990	0.948-1.034	0.703			
sTLG	0.999	0.995-1.003	0.686			
TLG	1.000	1.000-1.000	0.607			
MTV	1.000	0.999-1.001	0.588			

^†^ OR, odds ratio; UNI, univariate analysis; MULTI, multivariate analysis; CI, confidence interval; SUV max, maximal standardized uptake value; MTV, total metabolic tumor volume; SMTV, standardized total metabolic tumor volume; TLG, total lesion glycolysis; STLG, standardized total lesion glycolysis; D max, maximal inter-lesion distance; DEL, Double-Expressor Lymphoma.

### Habitat subregion construction and performance of the habitat radiomics models

**Figure 3:**
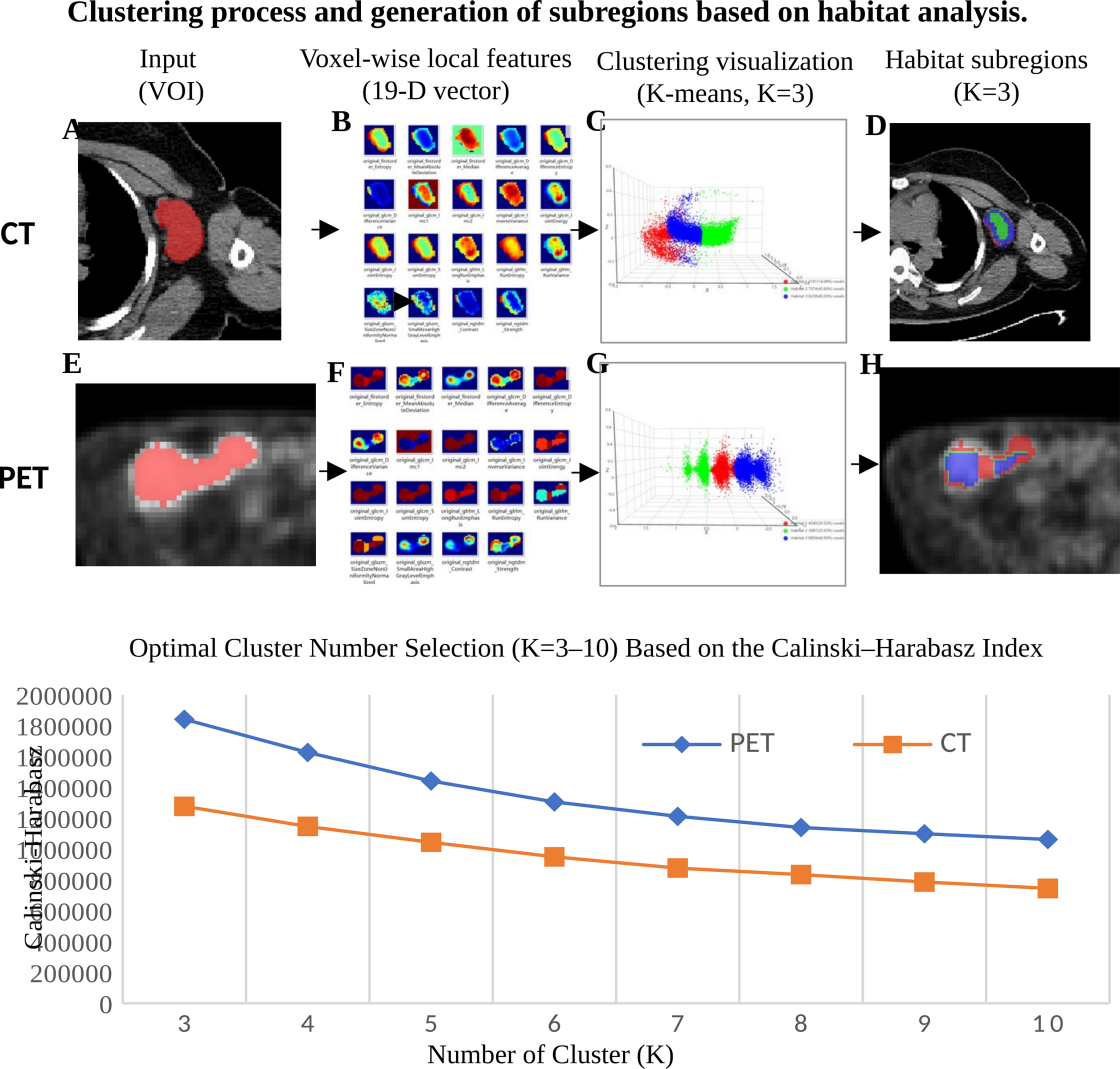
The process of clustering and generation of sub-regions based on habitat analysis technology. Habitat generation by voxel-wise clustering for CT **(A–D)** and PET **(E–H)**. The tumor VOI **(A, E)** was used to compute voxel-wise 19-dimensional local feature vectors **(B, F)**, followed by K-means clustering **(C, G)** to obtain three habitat subregions (K = 3) mapped back to the images **(D, H)**. The Calinski–Harabasz index across candidate cluster numbers (K = 3–10) is shown to support selection of K = 3.

During habitat construction, the Calinski–Harabasz (CH) index was used as the primary criterion for determining the optimal number of clusters, while the Silhouette score and Davies–Bouldin index were used as additional reference metrics. For both PET- and CT-derived habitat clustering, the CH index peaked at K = 3, as shown in **Figure 3**. The additional reference metrics also supported this configuration, with K = 3 showing the highest Silhouette score and the lowest Davies–Bouldin index for both PET and CT clustering. These concordant findings supported the selection of three habitat subregions and suggested favorable cluster compactness and separation.

Accordingly, three tumor habitat subregions were delineated separately on PET and CT images, and the corresponding whole-tumor region was retained for each modality. This yielded eight analysis regions per patient. From each habitat subregion and the whole-tumor region, 2016 PET radiomics features and 1834 CT radiomics features were extracted, yielding 15,400 features per patient (2,016+1,834)×(3subregions+1whole tumorregion).

**Figure 4:**
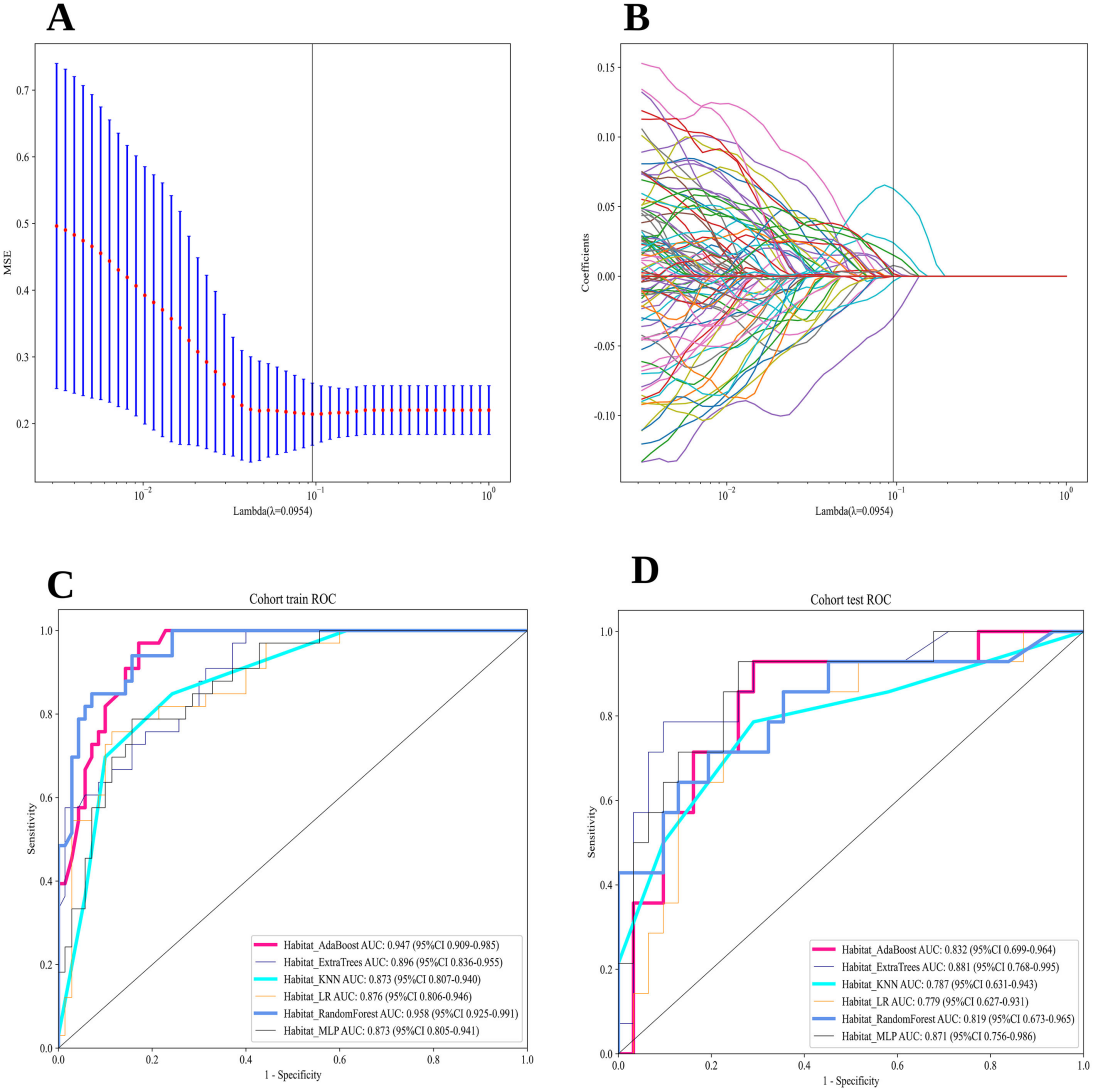
Radiomics feature selection and performance comparison of habitat-based models derived from baseline ¹^8^F-FDG PET/CT. **(A)** Ten-fold cross-validated mean squared error (MSE) versus log(λ) for LASSO. **(B)** LASSO coefficient paths. **(C, D)** ROC curves of habitat radiomics classifiers in the training **(C)** and testing **(D)** cohorts. ROC, receiver operating characteristic.

Following feature extraction, the inter-observer reproducibility of radiomic features was first assessed to ensure robustness against segmentation variability. A subset of 30 patients was randomly selected, and tumor VOIs were independently re-segmented by the two nuclear medicine physicians using the standardized protocol. The intraclass correlation coefficient (ICC) was calculated for all extracted features, and those with poor reproducibility (ICC < 0.80) were excluded. After feature selection, eight features with nonzero coefficients were retained (detailed feature names and coefficients are provided in **Supplementary Material 3**). Based on this feature set, multiple mainstream machine learning models were constructed and their performance was systematically evaluated in an independent test set, as shown in **Table 3**, **Figure 4**. Among these models, the Extra Trees achieved the highest AUC (0.881; 95% CI: 0.7678–0.9949). However, its performance was highly imbalanced, with very low sensitivity (0.071) and perfect specificity (1.000). This high-specificity/low-sensitivity pattern indicates a strong bias toward negative classification, resulting in poor identification of non-EMR patients and limited clinical utility. In contrast, the multilayer perceptron (MLP) provided the most robust and balanced performance in the test set. Although its AUC (0.871; 95% CI: 0.7563–0.9857) was slightly lower than that of Extra Trees, MLP achieved higher accuracy (0.822) and balanced accuracy (BACC = 0.773) with a better balance between sensitivity (0.643) and specificity (0.903), and a substantially higher F1 score (0.692 vs 0.133). MLP also outperformed Random Forest (AUC = 0.819; 95% CI: 0.6729–0.9653) and AdaBoost (AUC = 0.832; 95% CI: 0.6994–0.9642). These results indicate superior robustness of the MLP in handling class imbalance and greater suitability for clinically relevant risk stratification. Considering discrimination, class-balance, and clinical relevance, MLP was selected as the optimal habitat radiomics classifier for subsequent comparison with deep learning.

**Table 3:** Comparison of model performance for different machine learning algorithms based on radiomic features extracted from intratumoral and tumor subregional analyses.

Model name	ACC	BACC	AUC	95% CI	Sensitivity	Specificity	PPV	NPV	Precision	Recall	F1	Threshold	Cohort
LR	0.806	0.753	0.876	0.8063-0.9461	0.606	0.900	0.741	0.829	0.741	0.606	0.667	0.423	Train-label
LR	0.778	0.721	0.779	0.6269-0.9307	0.571	0.871	0.667	0.818	0.667	0.571	0.615	0.470	Test-label
KNN	0.835	0.799	0.873	0.8067-0.9400	0.697	0.900	0.767	0.863	0.767	0.697	0.730	0.400	Train-label
KNN	0.778	0.702	0.787	0.6312-0.9425	0.500	0.903	0.700	0.800	0.700	0.500	0.583	0.400	Test-label
RandomForest	0.883	0.834	0.958	0.9245-0.9910	0.697	0.971	0.920	0.872	0.920	0.697	0.793	0.307	Train-label
RandomForest	0.756	0.666	0.819	0.6729-0.9653	0.429	0.903	0.667	0.778	0.667	0.429	0.522	0.371	Test-label
ExtraTrees	0.767	0.637	0.896	0.8361-0.9552	0.273	1.000	1.000	0.745	1.000	0.273	0.429	0.254	Train-label
ExtraTrees	0.711	0.536	0.881	0.7678-0.9949	0.071	1.000	1.000	0.705	1.000	0.071	0.133	0.366	Test-label
AdaBoost	0.874	0.859	0.947	0.9089-0.9850	0.818	0.900	0.794	0.913	0.794	0.818	0.806	0.471	Train-label
AdaBoost	0.756	0.705	0.832	0.6994-0.9642	0.571	0.839	0.615	0.812	0.615	0.571	0.593	0.466	Test-label
MLP	0.786	0.707	0.873	0.8052-0.9411	0.485	0.929	0.762	0.793	0.762	0.485	0.593	0.393	Train-label
MLP	0.822	0.773	0.871	0.7563-0.9857	0.643	0.903	0.750	0.848	0.750	0.643	0.692	0.338	Test-label

^†^ ACC, accuracy; BACC, balanced accuracy; AUC, the area under the receiver operating characteristic curve; PPV, positive predictive value; NPV, negative predictive value; F1, F1 Score.

### Performance of the dual-channel deep learning models

**Figure 5:**
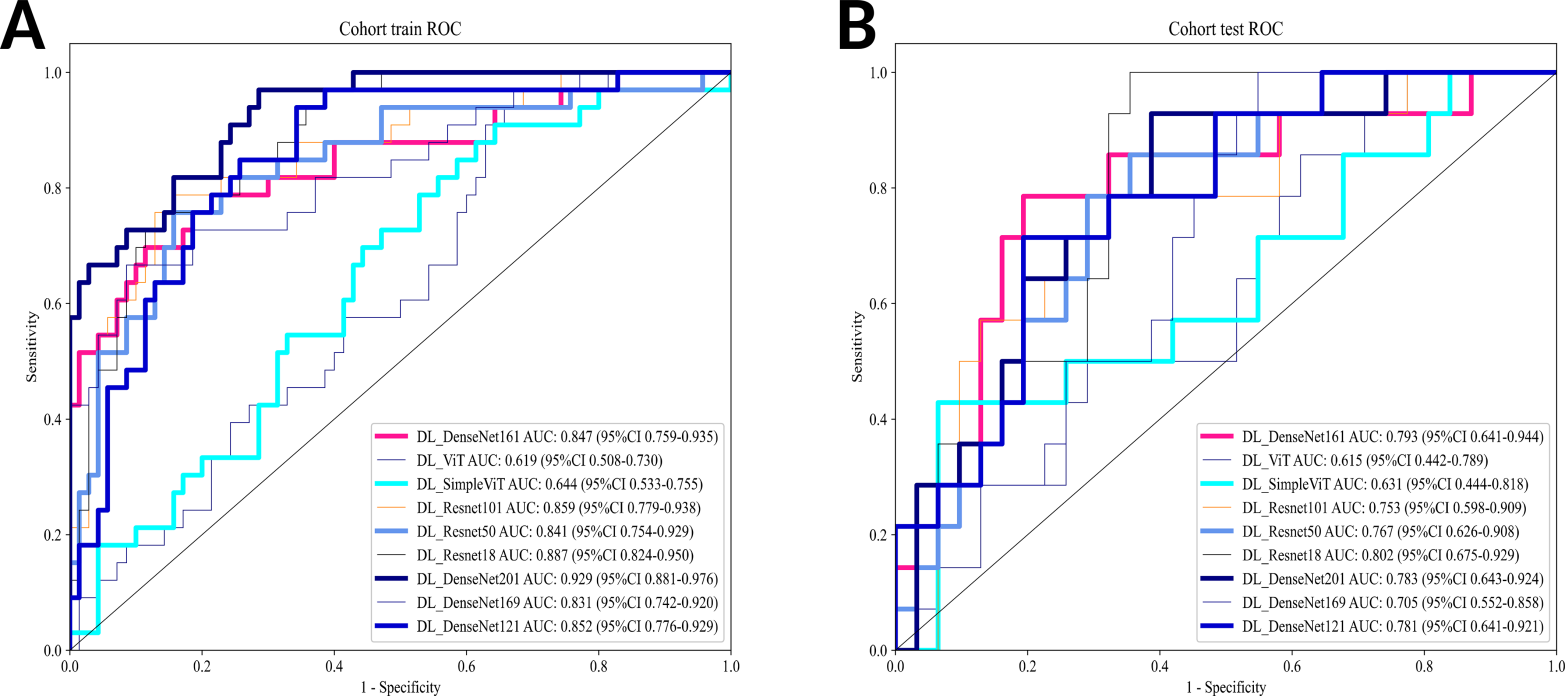
ROC curves of the evaluated dual-channel deep learning (DL) architectures for predicting interim PET early metabolic response (EMR) using baseline ¹^8^F-FDG PET/CT. **(A)** Training cohort ROC curves. **(B)** Testing cohort ROC curves.

During the construction of the dual-channel deep learning model, this study systematically compared the performance of nine different network architectures in predicting EMR in patients with DLBCL, including models from the ResNet, DenseNet, and Vision Transformer families. Model performance was primarily assessed using test-set AUC, together with the generalization gap (Gap), composite score (Score), and classification metrics (accuracy, sensitivity, and specificity) to gauge robustness and overfitting risk, as shown in **Table 4**, **Figure 5**. Among these architectures, the DenseNet161 architecture exhibited the best overall performance. DenseNet161 achieved a test AUC of 0.793 (95% CI: 0.6409–0.9444) and a training AUC of 0.847 (95% CI: 0.7585–0.9350), resulting in a relatively small generalization gap (Gap = 0.054) and the highest composite score (Score = 0.739). In the test set, DenseNet161 also showed relatively balanced classification (ACC = 0.711, BACC = 0.732, SEN = 0.786, SPE = 0.677). In contrast, although ResNet18 achieved the highest test set AUC (0.802; 95% CI: 0.6746–0.9291), its higher training AUC (0.887; 95% CI: 0.8243–0.9498) resulted in a larger gap (Gap = 0.085) and a lower composite score (Score = 0.717). ResNet18 also showed a high-sensitivity/low-specificity pattern (SEN = 1.000, SPE = 0.419), suggesting limited ability to identify negative cases and potential overfitting. In addition, transformer-based models showed lower discrimination and less stable feature learning. Therefore, DenseNet161 was selected as the optimal dual-channel model for subsequent comparison with habitat radiomics.

**Table 4:** Comparison of predictive performance across different network architectures in a dual-channel PET/CT deep learning model.

Model name	Acc	BACC	AUC	95% CI	Sensitivity	Specificity	PPV	NPV	Precision	Recall	F1	Threshold	Cohort	Gap	Score
DL_DenseNet161	0.825	0.736	0.847	0.7585-0.9350	0.485	0.986	0.941	0.802	0.941	0.485	0.640	0.385	train	0.054	0.739
DL_DenseNet161	0.711	0.732	0.793	0.6409-0.9444	0.786	0.677	0.524	0.875	0.524	0.786	0.629	0.638	test		
DL_ViT	0.641	0.576	0.619	0.5082-0.7299	0.394	0.757	0.433	0.726	0.433	0.394	0.413	0.221	train	0.004	0.611
DL_ViT	0.467	0.574	0.615	0.4416-0.7888	0.857	0.290	0.353	0.818	0.353	0.857	0.500	0.695	test		
DL_SimpleViT	0.680	0.500	0.644	0.5328-0.7546	0.000	1.000	0.000	0.680	0.000	0.000	—	0.308	train	0.012	0.619
DL_SimpleViT	0.689	0.500	0.631	0.4443-0.8184	0.000	1.000	0.000	0.689	0.000	0.000	—	0.300	test		
DL_Resnet101	0.806	0.761	0.859	0.7795-0.9383	0.636	0.886	0.724	0.838	0.724	0.636	0.677	0.384	train	0.105	0.648
DL_Resnet101	0.733	0.689	0.753	0.5979-0.9090	0.571	0.806	0.571	0.806	0.571	0.571	0.571	0.376	test		
DL_Resnet50	0.786	0.739	0.841	0.7537-0.9285	0.606	0.871	0.690	0.824	0.690	0.606	0.645	0.358	train	0.074	0.693
DL_Resnet50	0.689	0.677	0.767	0.6262-0.9084	0.643	0.710	0.500	0.815	0.500	0.643	0.562	0.333	test		
DL_Resnet18	0.786	0.683	0.887	0.8243-0.9498	0.394	0.971	0.867	0.773	0.867	0.394	0.542	0.246	train	0.085	0.717
DL_Resnet18	0.600	0.710	0.802	0.6746-0.9291	1.000	0.419	0.437	1.000	0.437	1.000	0.609	0.790	test		
DL_DenseNet201	0.854	0.805	0.929	0.8809-0.9762	0.667	0.943	0.846	0.857	0.846	0.667	0.746	0.167	train	0.145	0.638
DL_DenseNet201	0.689	0.716	0.783	0.6428-0.9240	0.786	0.645	0.500	0.870	0.500	0.786	0.611	0.351	test		
DL_DenseNet169	0.806	0.737	0.831	0.7420-0.9204	0.545	0.929	0.783	0.812	0.783	0.545	0.643	0.421	train	0.126	0.579
DL_DenseNet169	0.578	0.694	0.705	0.5519-0.8582	1.000	0.387	0.424	1.000	0.424	1.000	0.596	0.615	test		
DL_DenseNet121	0.777	0.700	0.852	0.7757-0.9290	0.485	0.914	0.727	0.790	0.727	0.485	0.582	0.217	train	0.071	0.710
DL_DenseNet121	0.622	0.687	0.781	0.6415-0.9207	0.857	0.516	0.444	0.889	0.444	0.857	0.585	0.792	test		

^†^ ACC, accuracy; BACC, balanced accuracy; AUC, the area under the receiver operating characteristic curve; PPV, positive predictive value; NPV, negative predictive value; F1, F1 Score; Gap, the generalization gap(Gap = AUC-train – AUC-test); Score, composite score (Score = AUC-test − α × (AUC-train – AUC-test)) α=1, —, no positive predictions/undefined.

### Comparative evaluation of model performance

**Figure 6:**
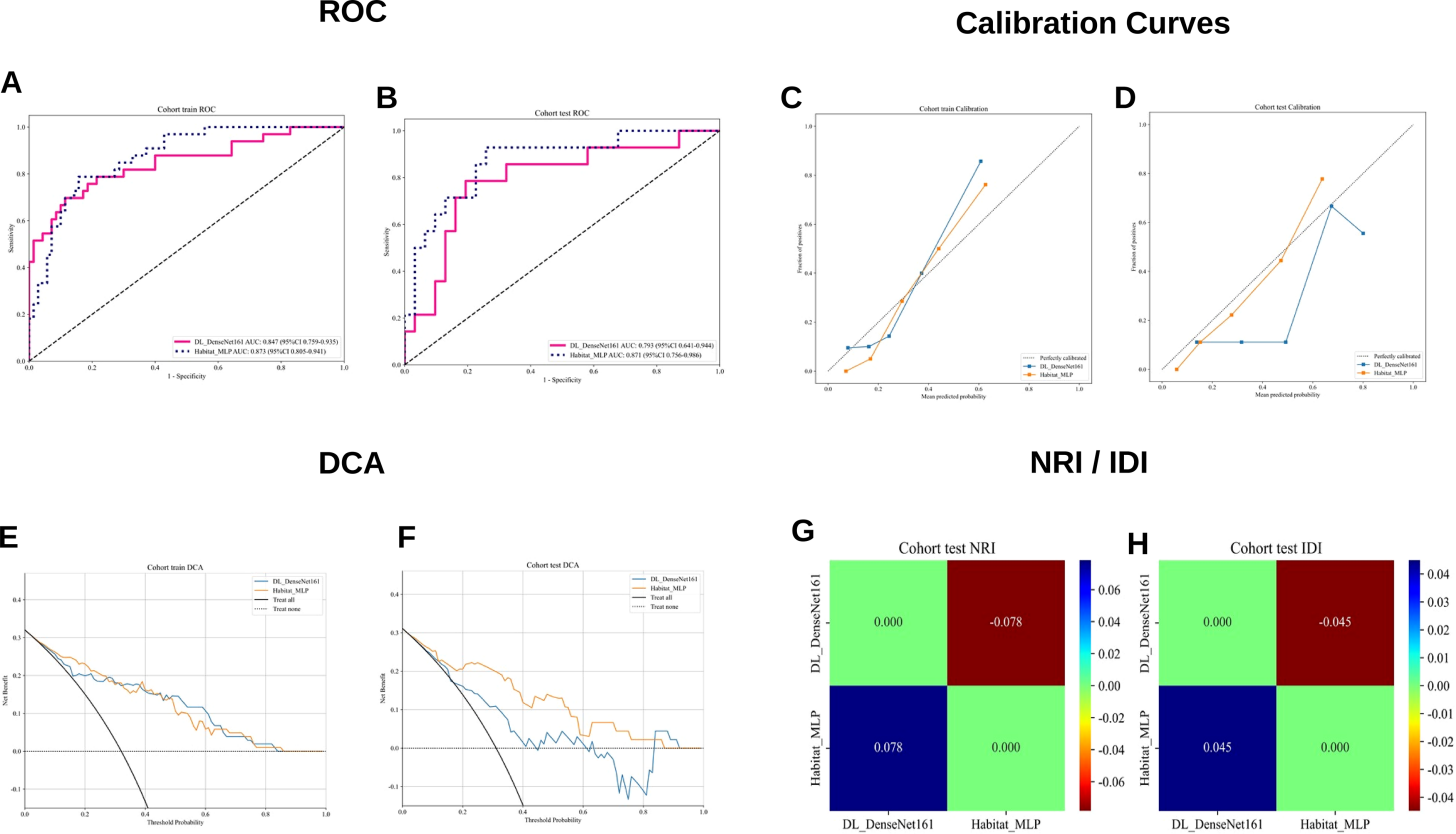
Comprehensive performance comparison between the habitat radiomics model (Habitat_MLP) and the dual-channel deep learning model (DL_DenseNet161) for predicting interim PET early metabolic response (EMR) in patients with DLBCL. **(A, B)** ROC curves in the training **(A)** and testing **(B)** cohorts; **(C, D)** calibration curves in the training **(C)** and testing **(D)** cohorts; **(E, F)** decision curve analysis in the training **(E)** and testing **(F)** cohorts; and **(G, H)** reclassification heatmaps in the testing cohort showing NRI **(G)** and IDI **(H)**.

We systematically compared Habitat_MLP and DL_DenseNet161 for predicting EMR in DLBCL in the training and test sets (**Figure 6**). In the training set, Habitat_MLP achieved a higher AUC (0.873; 95% CI: 0.8052–0.9411) than DL_DenseNet161 (0.847; 95% CI: 0.7585–0.9350). Notably, although DL_DenseNet161 showed higher training-set accuracy (0.825 vs 0.786), this advantage was not preserved in the independent test set, suggesting potential overfitting. In the independent test set, Habitat_MLP again achieved a higher AUC (0.871; 95% CI: 0.7563–0.9857) than DL_DenseNet161 (0.793; 95% CI: 0.6409–0.9444). Habitat_MLP also showed stronger generalization, with minimal AUC difference between training and test sets (0.873 vs 0.871). In addition, Habitat_MLP yielded higher accuracy (0.822 vs 0.711), balanced accuracy (0.773 vs. 0.732), specificity (0.903 vs 0.677), and F1 score (0.692 vs 0.629). Although DL_DenseNet161 showed slightly higher sensitivity (0.786 vs 0.643), its lower specificity indicates a higher false-positive rate, which may reduce clinical usefulness for risk stratification. To further explore differences between the two models, additional multidimensional evaluations were performed. Calibration curve analysis demonstrated good agreement between predicted and observed outcomes for both models in the training and test sets, with closer alignment to the ideal calibration curve observed for the Habitat_MLP in the test set. DCA showed that, across a wide range of threshold probabilities, the Habitat_MLP provided higher and more stable net benefit, whereas the net benefit of the DL_DenseNet161 declined in certain mid-to-high threshold ranges. Reclassification analysis further supported these findings: compared with the DL_DenseNet161, the Habitat_MLP achieved positive NRI (NRI = 0.078) and IDI (IDI = 0.045) in the test set, indicating superior risk stratification capability. Overall, the habitat radiomics model (Habitat_MLP) demonstrated higher predictive performance across multiple evaluation metrics than the deep learning model (DL_DenseNet161).

## Discussion

In the clinical management of DLBCL, iPET is widely used to assess EMR and is closely associated with patient prognosis (19). However, because iPET is performed during therapy, it offers limited guidance for pretreatment risk stratification and individualized decision-making. Accordingly, this study developed habitat radiomics and dual-channel deep learning models based on baseline ¹^8^F-FDG PET/CT to predict EMR status on iPET and compared their performance using the same dataset and evaluation framework. Both approaches showed acceptable discrimination; however, Habitat_MLP achieved higher performance, stronger generalization, and better class-balance in this study.

From an imaging biology perspective, DLBCL is a highly heterogeneous and aggressive lymphoma, with marked intratumoral differences in metabolic activity, cellular density, and the tumor microenvironment. This spatial heterogeneity is increasingly recognized as a key factor that may influence chemosensitivity and EMR (20). Conventional whole-tumor radiomics often treats the tumor as a homogeneous region, which can introduce an averaging effect and obscure localized information relevant to response. To address this limitation, we used voxel-wise habitat partitioning: intratumoral voxels with similar imaging characteristics were clustered into subregions, and region-specific features were extracted to better capture spatial heterogeneity (12, 14). In our data, the habitat radiomics model showed stable performance and favorable risk stratification for EMR prediction. It should be noted that habitat subregions do not directly correspond to specific molecular or pathological subtypes but should instead be regarded as imaging surrogates. Differences in metabolic intensity and textural patterns captured by these habitats may indirectly reflect distinct intratumoral microenvironmental states or clonal distributions, which may partially explain their association with treatment response (21).

We also evaluated a dual-channel deep learning model for EMR prediction on iPET. The model adopted an early PET/CT dual-channel fusion strategy, which leverages the complementary information of the two modalities while maintaining model trainability. In DLBCL, CT provides high-resolution anatomical and textural information (e.g., density changes and necrosis-related features), whereas PET reflects tumor glucose metabolism and metabolic heterogeneity (22, 23). By extracting the largest axial tumor slice within the VOI and concatenating PET and CT images along the channel dimension, the network can establish pixel-wise cross-modal correspondences early in feature learning. Compared with late-fusion strategies that combine modality-specific features only after separate extraction, this approach may better capture integrated metabolic–anatomical patterns (24).

In this study, the dual-channel deep learning model did not outperform habitat radiomics. This does not indicate an inherent limitation of deep learning; rather, it may reflect a mismatch among dataset size, task complexity, and model capacity. Deep learning typically benefits from large datasets to learn stable and generalizable high-dimensional representations, whereas in single-center studies with limited sample sizes, performance can be more vulnerable to overfitting (25, 26). In contrast, habitat radiomics models, which rely on manually engineered features combined with rigorous feature selection procedures, tend to exhibit greater robustness and interpretability in small- to moderate-sized datasets (27, 28). This observation is consistent with findings from previous imaging artificial intelligence studies, which emphasize that model complexity should be appropriately matched to data scale (29).

From a clinical application perspective, predictive models are required not only to achieve high discriminative performance but also to meet practical requirements in terms of classification balance, calibration consistency, and net benefit. In our analyses, the habitat radiomics model showed more stable calibration and decision-curve performance and yielded higher net benefit across a broader range of threshold probabilities. Positive NRI and IDI further suggested improved patient risk stratification relative to the deep learning model. Collectively, these findings suggest that, within the current study setting, the habitat radiomics model better meets the fundamental requirements of a clinically useful decision-support tool.

This study systematically compared two imaging analysis strategies—habitat radiomics models and dual-channel deep learning models—and comprehensively evaluated their predictive performance and potential clinical utility using multiple metrics. Although both models showed some ability to predict EMR on iPET in patients with DLBCL, several limitations should be acknowledged. First, this was a retrospective, single-center study; therefore, selection bias cannot be ruled out, and the generalizability of our findings requires further confirmation. In particular, an independent external validation set could not be obtained because of institutional data privacy restrictions. To provide an internal estimate of model performance, we used an internal hold-out test strategy in which the test set was kept completely independent from feature selection and model training. In addition, ICC-based reproducibility assessments were performed to improve feature robustness against segmentation variability. Nevertheless, good retrospective performance does not necessarily translate into real-world clinical utility. Therefore, before clinical adoption, the model should be validated in prospective, multicenter studies using a pre-specified protocol and a locked model to confirm its reproducibility and generalizability across different PET/CT scanners, reconstruction settings, and patient populations. Second, although a stratified 7:3 train/test split was used to maintain similar EMR/non-EMR proportions between the training and test sets, the use of a single random split may still introduce split-dependent variability in model performance. Repeated cross-validation, bootstrapping, or permutation-based validation was not performed in the present study. Future studies with larger multicenter datasets should incorporate repeated resampling-based validation strategies to further assess model robustness and generalizability. Third, the inherent “black-box” nature of deep learning models may limit clinical interpretability. Therefore, incorporating more transparent and explainable artificial intelligence approaches may help enhance clinical trust.

Fourth, although habitat analysis quantifies imaging heterogeneity, habitat delineation depends on the clustering method and parameter settings. Further work is needed to assess the robustness and reproducibility of habitat partitioning and to promote methodological standardization. In addition, although KNN imputation was used to handle missing habitat-specific radiomics features by preserving patient-level similarity in the multivariate feature space, we acknowledge that a formal comparison with alternative imputation strategies was not performed in this study. Because different imputation strategies may influence feature distributions, feature selection, and model robustness, future studies with larger multicenter datasets should further evaluate the sensitivity of habitat radiomics models to different missing-value imputation methods.

## Conclusion

In conclusion, this study developed and validated a habitat radiomics model (Habitat_MLP) and systematically compared it with a dual-channel deep learning model under the same prediction task. Habitat_MLP showed superior overall performance and greater stability for predicting EMR in patients with DLBCL, suggesting potential value as an imaging-based marker for pretreatment risk assessment.

## Data Availability

The data analyzed in this study is subject to the following licenses/restrictions: The dataset is derived from hospital medical records and contains sensitive patient information. Therefore, it is not publicly available due to patient privacy, confidentiality, and institutional/ethical restrictions. Requests to access these datasets should be directed to Ying Kou, KY596976312@163.com, and Zhuzhong Cheng, chengzhuzhong@scszlyy.org.cn.

## References

[1] Swerdlow SH Campo E Pileri SA Harris NL Stein H Siebert R . The 2016 revision of the World Health Organization classification of lymphoid neoplasms. Blood. (2016) 127:2375–90. doi: 10.1182/blood-2016-01-643569. PMID: 26980727 PMC4874220

[2] Sehn LH Salles G . Diffuse large B-cell lymphoma. N Engl J Med. (2021) 384:842–58. doi: 10.1016/j.hoc.2016.07.010. PMID: 33657296 PMC8377611

[3] Coiffier B Lepage E Briere J Herbrecht R Tilly H Bouabdallah R . CHOP chemotherapy plus rituximab compared with CHOP alone in elderly patients with diffuse large-B-cell lymphoma. N Engl J Med. (2002) 346:235–42. doi: 10.1056/nejmoa011795. PMID: 11807147

[4] Sehn LH Gascoyne RD . Diffuse large B-cell lymphoma: optimizing outcome in the context of clinical and biologic heterogeneity. Blood. (2015) 125:22–32. doi: 10.1182/blood-2014-05-577189. PMID: 25499448

[5] Cheson BD Fisher RI Barrington SF Cavalli F Schwartz LH Zucca E . Recommendations for initial evaluation, staging, and response assessment of Hodgkin and non-Hodgkin lymphoma: the Lugano classification. J Clin Oncol. (2014) 32:3059–68. doi: 10.1200/jco.2013.54.8800. PMID: 25113753 PMC4979083

[6] Mamot C Klingbiel D Hitz F Renner C Pabst T Driessen C . Final results of a prospective evaluation of the predictive value of interim positron emission tomography in patients with diffuse large B-cell lymphoma treated with R-CHOP-14 (SAKK 38/07). J Clin Oncol. (2015) 33:2523–9. doi: 10.1200/jco.2014.58.9846. PMID: 26150440

[7] Kurch L Hüttmann A Georgi TW Rekowski J Sabri O Schmitz C . Interim PET in diffuse large B-cell lymphoma. J Nucl Med. (2021) 62:1068–74. doi: 10.2967/jnumed.120.255034. PMID: 33246974

[8] Barrington SF Mikhaeel NG Kostakoglu L Meignan M Hutchings M Müeller SP . Role of imaging in the staging and response assessment of lymphoma: consensus of the International Conference on Malignant Lymphomas Imaging Working Group. J Clin Oncol. (2014) 32:3048–58. doi: 10.1200/jco.2013.53.5229. PMID: 25113771 PMC5015423

[9] Lambin P Rios-Velazquez E Leijenaar R Carvalho S van Stiphout RGPM Granton P . Radiomics: extracting more information from medical images using advanced feature analysis. Eur J Cancer. (2012) 48:441–6. doi: 10.1016/j.ejca.2011.11.036. PMID: 22257792 PMC4533986

[10] Carlier T Frécon G Mateus D Rizkallah M Kraeber-Bodéré F Kanoun S . Prognostic value of ^18^F-FDG PET radiomics features at baseline in PET-guided consolidation strategy in diffuse large B-cell lymphoma: a machine-learning analysis from the GAINED study. J Nucl Med. (2024) 65:156–62. doi: 10.1007/s00701-025-06710-5. PMID: 37945379

[11] Eertink JJ Zwezerijnen GJC Heymans MW Pieplenbosch S Wiegers SE Dührsen U . Baseline PET radiomics outperforms the IPI risk score for prediction of outcome in diffuse large B-cell lymphoma. Blood. (2023) 141:3055–64. doi: 10.1182/blood.2022018558. PMID: 37001036 PMC10646814

[12] Gatenby RA Grove O Gillies RJ . Quantitative imaging in cancer evolution and ecology. Radiology. (2013) 269:8–15. doi: 10.1148/radiol.13122697. PMID: 24062559 PMC3781355

[13] Li S Dai Y Chen J Yan F Yang Y . MRI-based habitat imaging in cancer treatment: current technology, applications, and challenges. Cancer Imaging. (2024) 24:107. doi: 10.1186/s40644-024-00758-9. PMID: 39148139 PMC11328409

[14] Zhang X Su GH Chen Y Gu YJ You C . Decoding intratumoral heterogeneity: clinical potential of habitat imaging based on radiomics. Radiology. (2023) 309:e232047. doi: 10.1148/radiol.232047. PMID: 38085080

[15] Esteva A Robicquet A Ramsundar B Kuleshov V DePristo M Chou K . A guide to deep learning in healthcare. Nat Med. (2019) 25:24–9. doi: 10.1038/s41591-018-0316-z. PMID: 30617335

[16] Litjens G Kooi T Bejnordi BE Setio AAA Ciompi F Ghafoorian M . A survey on deep learning in medical image analysis. Med Img Anal. (2017) 42:60–88. doi: 10.1016/j.media.2017.07.005. PMID: 28778026

[17] Shiri I Amini M Yousefirizi F Vafaei Sadr A Hajianfar G Salimi Y . Information fusion for fully automated segmentation of head and neck tumors from PET and CT images. Med Phys. (2024) 51:319–33. doi: 10.1002/mp.16615. PMID: 37475591

[18] Ma B Guo J Dijk LV Langendijk JA Ooijen PMA Both S . PET and CT based DenseNet outperforms advanced deep learning models for outcome prediction of oropharyngeal cancer. Radiother Oncol. (2025) 207:110852. doi: 10.1016/j.radonc.2025.110852. PMID: 40118186

[19] Rekowski J Hüttmann A Schmitz C Müller SP Kurch L Kotzerke J . Interim PET evaluation in diffuse large B-cell lymphoma using published recommendations: comparison of the Deauville 5-point scale and the ΔSUVmax method. J Nucl Med. (2021) 62:37–42. doi: 10.2967/jnumed.120.255034. PMID: 32385164

[20] Jain RK . Normalizing tumor microenvironment to treat cancer: bench to bedside to biomarkers. J Clin Oncol. (2013) 31:2205–18. doi: 10.1200/jco.2012.46.3653. PMID: 23669226 PMC3731977

[21] Chen H Liu Y Zhao J Jia X Chai F Peng Y . Quantification of intratumoral heterogeneity using habitat-based MRI radiomics to identify HER2-positive, -low and -zero breast cancers: a multicenter study. Breast Cancer Res. (2024) 26:160. doi: 10.1186/s13058-024-01921-7. PMID: 39578913 PMC11583526

[22] Reinert CP Perl RM Faul C Lengerke C Nikolaou K Dittmann H . Value of CT-textural features and volume-based PET parameters in comparison to serologic markers for response prediction in patients with diffuse large B-cell lymphoma undergoing CD19-CAR-T cell therapy. J Clin Med. (2022) 11:1522. doi: 10.3390/jcm11061522. PMID: 35329846 PMC8951429

[23] Xin W Wang F Gu W Wang Y . Association analysis of intratumoral metabolic heterogeneity assessed by the hottest lesion based on ^18^F-FDG PET/CT with immunochemotherapy response in diffuse large B-cell lymphoma. Quant Imaging Med Surg. (2025) 15:8096–111. doi: 10.21037/qims-2024-2699. PMID: 40893516 PMC12397664

[24] Huang SC Pareek A Seyyedi S Banerjee I Lungren MP . Fusion of medical imaging and electronic health records using deep learning: a systematic review and implementation guidelines. NPJ Dig Med. (2020) 3:136. doi: 10.1038/s41746-020-00341-z. PMID: 33083571 PMC7567861

[25] Samala RK Chan HP Hadjiiski L Helvie MA . Risks of feature leakage and sample size dependencies in deep feature extraction for breast mass classification. Med Phys. (2021) 48:2827–37. doi: 10.1002/mp.14678. PMID: 33368376 PMC8601676

[26] Zantvoort K Nacke B Görlich D Hornstein S Jacobi C Funk B . Estimation of minimal data sets sizes for machine learning predictions in digital mental health interventions. NPJ Dig Med. (2024) 7:361. doi: 10.1038/s41746-024-01360-w. PMID: 39695276 PMC11655521

[27] Rundo L Militello C . Image biomarkers and explainable AI: handcrafted features versus deep learned features. Eur Radiol Exp. (2024) 8:130. doi: 10.1186/s41747-024-00529-y. PMID: 39560820 PMC11576747

[28] Buvat I Dutta J Jha AK Siegel E Yousefirizi F Rahmim A . Should end-to-end deep learning replace handcrafted radiomics? Eur J Nucl Med Mol Imaging. (2025) 52:4360–3. doi: 10.1007/s00259-025-07314-y. PMID: 40314811 PMC12491089

[29] Maier-Hein L Eisenmann M Reinke A Onogur S Stankovic M Scholz P . Why rankings of biomedical image analysis competitions should be interpreted with care. Nat Commun. (2018) 9:5217. doi: 10.1038/s41467-018-07619-7. PMID: 30523263 PMC6284017

