# Supplementary material for: Baseline 18F-FDG PET/CT habitat radiomics versus dual-channel deep learning for predicting interim PET early metabolic response in diffuse large B-cell lymphoma: a comparative study
Source: Front Oncol. 2026 Jun 4;16:1801204. doi: 10.3389/fonc.2026.1801204 (PMC13275354; doi:10.3389/fonc.2026.1801204)
Supplement: Supplementary file 1 [file DataSheet1.docx]

**Supplemental Material 1**

PET/CT imaging was conducted using a Siemens Biograph mCT-64 scanner. The radiotracer employed was 18F-fluorodeoxyglucose (18F-FDG), synthesized using a Sumitomo HM-10HC medical cyclotron and synthesis module (Sumitomo, Japan), with a radiochemical purity exceeding 99%. Prior to imaging, all patients underwent a fasting period, and their blood glucose levels were confirmed to be below 11.1 mmol/L. The administered radiotracer dose ranged from 3.7 to 5.5 MBq/kg, delivered via intravenous injection.

Standard gastrointestinal preparation involved oral administration of 1000 mL of a 1.5% diatrizoate meglumine solution. Of this, 800 mL was consumed evenly during the 50-minute radiotracer uptake period, while the remaining 200 mL was ingested immediately before scanning. Patients were instructed to remove metallic objects and empty their bladder prior to the examination.

A low-dose CT scan was initially performed for attenuation correction and image fusion, using a tube voltage of 120 kV and an effective tube current of 80 mAs. For PET acquisition, the scan duration was set to 1.5 minutes per bed position for the body (typically 6–7 bed positions depending on the patient’s height) and 2 minutes per bed position for the head.

Image reconstruction was conducted using a high-definition iterative algorithm combined with time-of-flight technology (TrueX + TOF, ultraHD-PET). The reconstruction parameters were set to 3 iterations and 21 subsets for the body and 4 iterations and 25 subsets for the head. Image fusion and interpretation were performed immediately following image acquisition.

**Supplemental Material 2.**

This study extracted 19 localized feature vectors: original_firstorder_Entropy, original_firstorder_MeanAbsoluteDeviation, original_firstorder_Median, original_glcm_DifferenceAverage, original_glcm_DifferenceEntropy, original_glcm_DifferenceVariance, original_glcm_Imc1, original_glcm_Imc2, original_glcm_InverseVariance, original_glcm_JointEnergy, original_glcm_JointEntropy, original_glcm_SumEntropy, original_glrlm_LongRunEmphasis, original_glrlm_RunEntropy, original_glrlm_RunVariance, original_glrlm_SizeZoneNonUniformityNormalized, original_glszm_SmallAreaHighGrayLevelEmphasis, original_ngtdm_Contrast, original_ngtdm_Strength.

**Supplemental Material 3.**

Eight radiomics features were retained after LASSO regression for the habitat models. These features, ranked by absolute coefficient magnitude, were:

(1) log_sigma_2_0_mm_3D_glcm_Idn_h3_x (coefficient: 0.0628);

(2) log_sigma_3_0_mm_3D_ngtdm_Busyness_h2_x (0.0241);

(3) wavelet_LHL_glszm_LargeAreaHighGrayLevelEmphasis_h3_y (0.0153);

(4) lbp_3D_m2_firstorder_Skewness_h3_y (0.0078);

(5) lbp_3D_m2_firstorder_Maximum_h1_x (0.0048);

(6) wavelet_LLH_firstorder_Skewness_h2_y (0.0018);

(7) wavelet_LHL_firstorder_Skewness_h3_y (−0.0035);

and (8) wavelet_LLH_glszm_SmallAreaEmphasis_h1_x (−0.0297).

Features were equally derived from CT (n = 4, suffix "_x") and PET (n = 4, suffix "_y"), with Habitat 3 contributing the most features (n = 4), followed by Habitat 1 and Habitat 2 (n = 2 each).
